# Nonspecific block of voltage‐gated potassium channels has greater effect on distal schaffer collaterals than proximal schaffer collaterals during periods of high activity

**DOI:** 10.14814/phy2.13354

**Published:** 2017-07-26

**Authors:** Benjamin Owen, Rishi Reddy, Lawrence M. Grover

**Affiliations:** ^1^ Department of Biomedical Sciences Marshall University School of Medicine Huntington West Virginia 25755; ^2^ Present address: Neuroscience Institute Morehouse School of Medicine Atlanta Georgia

**Keywords:** Axon excitability, burst stimulation, high‐frequency stimulation, voltage‐gated potassium channel

## Abstract

Previous studies established different responses between proximal and distal portions of Schaffer collateral axons during high‐frequency and burst stimulation, with distal axons demonstrating biphasic changes in excitability (hyperexcitability followed by depression), but proximal axons showing only monophasic depression. Voltage‐dependent potassium (K_V_) channels are important determinants of axonal excitability, and block of K_V_ channels can promote axon hyperexcitability. We therefore hypothesized that block of K_V_ channels should lead to biphasic response changes in proximal Schaffer collaterals, like those seen in distal Schaffer collaterals. To test this hypothesis, we made extracellular recordings of distal Schaffer collateral responses in stratum radiatum of hippocampal area CA1 and proximal Schaffer collateral responses in stratum pyramidale of area CA3 during high‐frequency stimulation (HFS) at 100 Hz and burst stimulation at 200 msec intervals (5 Hz or theta frequency). We then applied a nonselective K_V_ channel blocker, tetraethlylammonium (TEA, 10 mmol/L) or 4‐aminopyridine (4‐AP, 100 *μ*mol/L), and assessed effects on Schaffer collateral responses. Surprisingly, block of K_V_ channels had little or no effect on proximal Schaffer collateral responses during high‐frequency or burst stimulation. In contrast, K_V_ channel blockade caused more rapid depression of distal Schaffer collateral responses during both high‐frequency and burst stimulation. These findings indicate that K_V_ channels are important for maintaining distal, but not proximal, Schaffer collateral excitability during period of sustained high activity. Differential sensitivity of distal versus proximal Schaffer collaterals to K_V_ channel block may reflect differences in channel density, diversity, or subcellular localization.

## Introduction

The hippocampus, a brain region required for normal memory formation, has been used to study cellular mechanisms thought to underlie memory formation such as long‐term potentiation (LTP) of synaptic function (Bliss and Collingridge 1993; Blundon and Zakharenko 2008). Neurons of area CA3 are connected to those in CA1 via the Schaffer collaterals (Ishizuka et al. 1990; Li et al. 1994; Wittner et al. 2007), and form one component of the trisynaptic circuit of the hippocampus (O'Keefe and Nadel 1978; Andersen et al. 2000 ). The unmyelinated Schaffer collateral axons undergo changes in excitability during high‐frequency stimulation (HFS) and burst stimulation that vary with the frequency and pattern of activity, and that differ between distal and proximal locations (Kim et al. 2012; Owen and Grover 2015). However, it is unknown what mechanism(s) underlie these effects. One possibility is via the activity of voltage‐gated potassium (K_V_) channels.

There are 11 families of voltage‐gated potassium (K_V_) channels, but only those in the K_V_1‐4 and 7 families are fully independent and functional (Vacher et al. 2008). These families are associated with currents that regulate membrane potential and excitability, and loss of these currents is associated with increased neuronal activity. For example, pharmacological block of the D‐type current, which is associated with the K_V_1 family members (Bekkers and Delaney 2001; Bean 2007), increased neuronal firing and reduced firing threshold in layer 5 cortical pyramidal neurons (Bekkers and Delaney 2001). Nonspecific block of K_V_ channels by 4‐aminopyridine (4‐AP) has been shown to induce temporal lobe seizures in rats (Lévesque et al. 2013), and increase action potential firing and reduce firing threshold, in layer 5 cortical pyramidal neurons (Bekkers and Delaney 2001; Bean 2007). Specific K_V_ subunits vary in subcellular localization, with K_V_ 1, 2, 3, and 7 enriched on axons, axon initial segments, or axon terminals (Trimmer 2015). Pharmacological block of K_V_1 containing channels (Palani et al. 2010) facilitated bursting and hyperexcitability of Schaffer collateral axons. Similarly, genetic deletion of K_V_1 subunits (Lopantsev et al. 2003) resulted in CA3 pyramidal neuron burst firing in response to antidromic stimulation.

Previously, we reported that Schaffer collaterals undergo activity‐dependent changes in excitability, with distal portions of the axons showing biphasic changes (hyperexcitability followed by depression), while proximal axons only undergo depression (Kim et al. 2012; Owen and Grover 2015). These differences could be due to differences in density or type of K_V_ subunits expressed in proximal versus distal portions of Schaffer collaterals. Here, we examined the role of K_V_ channels in Schaffer collateral function using the nonspecific blockers tetraethylammonium (TEA) and 4‐AP. We hypothesized that K_V_ channel blockade would promote axon hyperexcitability and abolish the regional differences between distal and proximal axons. Surprisingly, nonselective block of K_V_ channels caused greater and more rapid depression of excitability in distal axons during high‐frequency and burst stimulation but had little effect on proximal axons.

## Materials and Methods

### Slice preparation

Hippocampal slices were prepared as previously described (Kim et al. 2012; Owen and Grover 2015). Male and female Sprague–Dawley rats (30–60 days old, Hilltop Lab Animals, Scottdale, PA) were sedated by CO_2_/air inhalation, and decapitated. The brain was removed and placed into chilled artificial cerebrospinal fluid (ACSF) composed of (in mmol/L): 124 NaCl, 26 NaHCO_3_, 3.4 KCl, 1.2 NaH_2_PO_4_, 2.0 CaCl_2_, 2.0 MgSO_4_, 10 glucose (pH 7.35, equilibrated with 95% O_2_/5% CO_2_). A block containing both hippocampi was glued to the stage of a vibratome (Campden Instruments, Lafayette, IN), immersed in chilled ACSF, and sectioned into 400–500 *μ*m thick slices in the coronal or horizontal plane. Slices were dissected to remove the hippocampus from surrounding structures. Hippocampal slices were stored at room temperature (20–22°C) in an interface holding chamber. For recordings, individual slices were transferred to a small volume (approximately 200 *μ*L) interface recording chamber heated to 34.5–35.5°C. Slices were perfused with oxygenated ACSF at a rate of 1–1.5 mL/min. All procedures were approved by the Institutional Animal Care and Use Committee at Marshall University.

### Field potential recording

**Figure 1:**
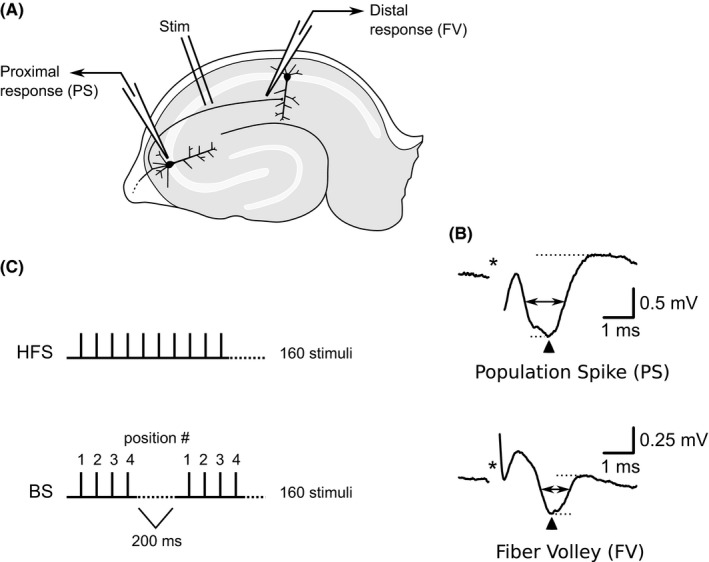
Illustration of stimulation and recording methods. (A) The stimulating electrode was placed in stratum radiatum near the border of areas CA3 and CA1. Simultaneous extracellular recordings were made from stratum pyramidale in area CA3 (population spike) and stratum radiatum in area CA1 (fiber volley). (B) Typical field potential responses recorded in DNQX (30 *μ*mol/L), to block AMPA recptors, and CGP‐37849 (5 *μ*mol/L), to block NMDA receptors. Stimulus artifacts (*) have been partially removed. Top: Population spike recorded from CA3 stratum pyramidale. Bottom: fiber volley recorded from CA1 stratum radiatum. Amplitudes were determined by the difference between the negative peak (dotted horizontal line) and following positive deflection (dotted horizontal line). Response latencies were determined by time difference between stimulation (*) and negative peak (upward pointing arrow head). Half‐widths were determined by response duration at half amplitude (double‐headed horizontal arrow). (C) Stimulation protocols. High‐frequency stimulation (HFS) consisted of 160 stimuli delivered at constant 10 msec intervals (100 Hz). Burst stimulation (BS) consisted of 160 stimuli delivered as short bursts of four stimuli at 10 msec intervals, with burst repeated every 200 msec (5 Hz). Stimulus position (1–4) within bursts is indicated by numerals.

Extracellular field potentials were recorded through glass micropipettes filled with ACSF (3–5 MΩ); in some recordings, the tip was broken prior to placement in the slice in order to lower resistance (1–2 MΩ) and reduce noise. Distal and proximal Schaffer collateral responses (fiber volleys and antidromic population spikes, respectively) were measured simultaneously by placing two recording electrodes in the slice: one in CA1 stratum radiatum and one in CA3 stratum pyramidale (Fig. 1A). Field potentials were recorded with an Axoclamp 2B using an HS‐2A headstage (Axon Instruments, now Molecular Devices) and an Ithaco 4302 dual filter unit, or a DAM50 (WPI) amplifier; potentials were band‐pass filtered at 0.1–3000 Hz and amplified with a gain of 1000. Signals were digitized (National Instruments PCI‐6035E or PCI‐1200) at 40–100 kHz and stored on a personal computer running Windows XP (Microsoft) using WinWCP and WinEDR software (Strathclyde Electrophysiology Software, John Dempster, University of Strathclyde). Fiber volley and population spike amplitude were measured as the difference between the maximum negativity and following positivity (Fig. 1B). Latencies were measured as the time difference between the beginning of the stimulus artifact and the response at peak negative amplitude. Half‐widths were measured as response duration at one‐half of the maximum amplitude.

### Stimulation

A bipolar, Teflon^®^ insulated, stainless steel stimulating electrode was placed in stratum radiatum near the border of areas CA3 and CA1. Constant voltage, biphasic stimuli (duration 0.1 msec) were delivered using an A‐M Systems model 2100 stimulator. The stimulus intensity was adjusted to produce the largest response which could be obtained without contamination of the response by the stimulus artifact. Stimulus intensities were typically 3–10 V. Three types of stimulus protocols were used: low‐frequency stimulation, continuous HFS, and burst stimulation (Fig. 1C). For low‐frequency stimulation, single stimuli were delivered once every 15 sec (0.067 Hz). For HFS, trains of 160 pulses at 100 Hz were delivered. For burst stimulation, bursts of four stimuli at 100 Hz were repeated at 200 msec intervals, for a total of 40 bursts (160 stimuli). Fiber volleys and population spikes were isolated using an AMPA receptor blocker (30 *μ*mol/L DNQX) and an NMDA receptor antagonist (5 *μ*mol/L CGP‐37849). For recordings made with 4‐AP, the GABA_A_ blocker, bicuculline (10 *μ*mol/L) was also applied.

### Reagents

Most drugs were prepared as concentrated stock solutions. 6,7‐dinitroquinoxaline‐2,3‐dione (DNQX 30 mmol/L; Tocris) was dissolved in dimethyl sulfoxide (DMSO), while CGP‐37849 (5 mmol/L, Tocris) bicuculline methiodide (10 mmol/L, Tocris) and 4‐AP (100 *μ*mol/L, Sigma) were dissolved in water. Stock solutions were diluted to final concentrations by addition to ACSF perfusing the tissue. TEA (10 mmol/L; chloride salt) was dissolved directly into ACSF, substituted on an equimolar basis for NaCl. Salts and all other reagents were from Sigma or Fisher.

### Data analysis and Statistics

Responses were analyzed for amplitude, latency, and half‐width; for comparison among slices, amplitudes were normalized relative to the first response recorded during each round of high‐frequency or burst stimulation. For statistical analysis, data were grouped by stimulus number: 3–7, 18–22, 38–42, and 156–160 for HFS; 1–4, 41–44, and 157–160 for burst stimulation. Statistical analysis was by paired t‐tests (where appropriate) or by repeated measures analysis of variance (ANOVA). A *P* ≤ 0.05 was accepted as significant; post hoc paired comparisons was made using the Bonferonni method. SPSS (IBM) and Gnumeric (http://www.gnome.org/projects/gnumeric/) were used for data analysis and statistical comparisons.

## Results

### Effects of nonspecific block of K_V_ channels on responses evoked during low‐frequency stimulation

**Figure 2:**
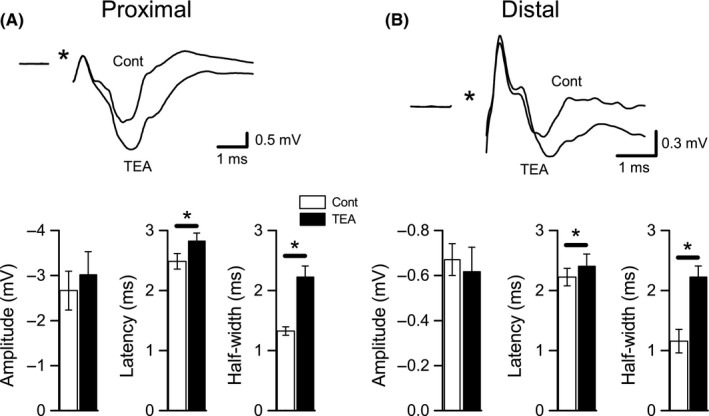
Effects of TEA on proximal and distal Schaffer collateral responses evoked at low frequency. (A) Effects on proximal Schaffer collateral responses (CA3 population spikes). Representative recording from a single slice is shown at top; control (Cont) responses were evoked at 0.067 Hz in DNQX (30 *μ*mol/L) + CGP‐37849 (5 *μ*mol/L) and averaged over a 5‐m period prior to TEA (10 mmol/L) application and over a 5‐min period after TEA was applied. Averaged measurements of proximal response amplitude, latency, and half‐width are shown at bottom. Latencies and half‐widths were significantly increased after TEA application, but amplitudes were not changed. (B) Effects on distal Schaffer collateral responses (CA1 fiber volleys). Responses shown at top were recorded from the same slice shown in A, at the same time points. Averaged measurements of distal response amplitude, latency, and half‐width are shown at bottom. As with proximal responses, distal latencies and half‐widths were significantly increased after TEA application, but amplitudes were not changed. *P < 0.05 (Cont vs. TEA).

**Figure 3:**
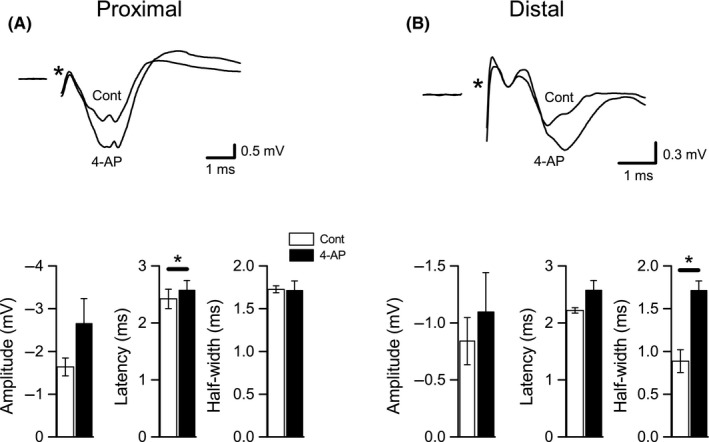
Effects of 4‐AP on proximal and distal Schaffer collateral responses evoked at low frequency. (A) Effects on proximal Schaffer collateral responses. Representative recording from a single slice is shown at top; control (Cont) responses were evoked at 0.067 Hz in DNQX (30 *μ*mol/L) + CGP‐37849 (5 *μ*mol/L) and averaged over a 5‐m period prior to 4‐AP (100 *μ*mol/L) application and over a 5‐min period after 4‐AP was applied. Averaged measurements of proximal response amplitude, latency, and half‐width are shown at bottom. Latencies were significantly increased after 4‐AP application, but amplitudes and half‐widths were not changed. B. Effects of 4‐AP on distal Schaffer collateral responses. Responses shown at top were recorded from the same slice shown in A, at the same time points. Averaged measurements of distal response amplitude, latency, and half‐width are shown at bottom. Half‐widths were significantly increased after 4‐AP application, but amplitudes and latencies were not changed. *P < 0.05 (Cont vs. 4‐AP).

Bath application of either TEA (10 mmol/L, *n* = 8) or 4‐AP (100 μmol/L, *n* = 6) led to prominent changes in both proximal and distal Schaffer collateral responses evoked during low‐frequency stimulation (Figs. 2, 3). Although we did not observe any significant changes in response amplitude, we did observe a significant increase in the latencies (control vs. TEA: 2.49 ± 0.13 msec vs. 2.83 ± 0.13 msec, *P* < 0.001) and half‐widths (control vs. TEA: 2.49 ± 0.13 msec vs. 2.83 ± 0.13 msec, *P* < 0.005) of proximal axon responses after applying TEA. Similarly, distal axon responses had longer latencies (control vs. TEA: 2.22 ± 0.15 msec vs. 2.41 ± 0.19 msec, *P* < 0.05) and half‐widths (control vs. TEA: 1.16 ± 0.2 msec vs. 1.81 ± 0.17 msec, *P* < 0.001) after TEA application. In contrast, 4‐AP had fewer effects on Schaffer collateral responses, with only a small, but significant, increase in latency of proximal axon responses (control vs. 4‐AP: 2.42 ± 0.17 msec vs. 2.58 ± 0.16 msec, *P* < 0.05) and a significant increase in half‐width of distal responses (control vs. 4‐AP: 0.89 ± 0.13 msec vs. 1.72 ± 0.11 msec, *P* < 0.005). These effects on low‐frequency‐evoked responses verify that the concentrations of TEA and 4‐AP we used were sufficient to at least partly block K_V_ channels and alter Schaffer collateral function. In the next set of experiments, we assessed the effects of the same concentrations of blockers on responses evoked during high‐frequency and burst stimulation.

### Nonspecific block of K_V_ channels altered distal, but not proximal, Schaffer collateral function during HFS

**Figure 4:**
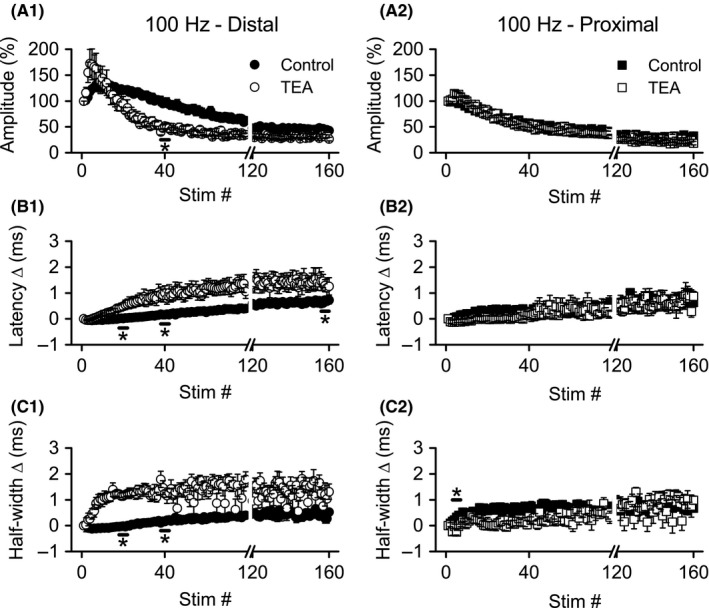
Application of TEA caused more rapid depression of distal, but not proximal, Schaffer collateral responses during 100 Hz HFS. Simultaneous recordings of distal (left) and proximal (right) Schaffer collateral responses during HFS were made in DNQX (30 *μ*mol/L) + CGP‐37849 (5 *μ*mol/L) before (Control) and after addition of TEA (10 mmol/L). Mean response amplitudes (A), latency changes (B), and half‐widths (C) were measured during HFS (*n* = 8): distal responses were greatly altered, but proximal responses were minimally affected. *P < 0.05 (Cont vs. TEA). For clarity, error bars are shown only for every fourth response. Data are not shown for stimuli 80–119 (break in *x*‐axis). HFS, high‐frequency stimulation.

**Figure 5:**
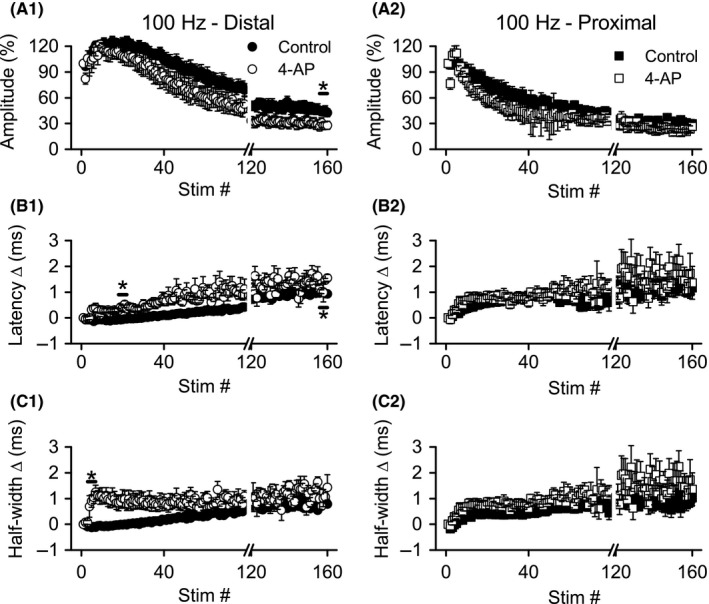
Application of 4‐AP caused more rapid depression of distal, but not proximal, Schaffer collateral responses during 100 Hz HFS. Simultaneous recordings of distal (left) and proximal (right) Schaffer collateral responses during HFS were made in DNQX (30 *μ*mol/L) + CGP‐37849 (5 *μ*mol/L) before (Control) and after addition of 4‐AP (100 *μ*mol/L). Mean response amplitudes (A), latency changes (B) and half‐widths (C) measured during HFS (*n* = 6): distal, but not proximal, responses were significantly affected. *P < 0.05 (Cont vs. 4‐AP). For clarity, error bars are shown only for every fourth response. Data are not shown for stimuli 80–119 (break in *x*‐axis).

We compared response amplitudes, latency and half‐width changes, at four time points during HFS (during responses 3–7, 18–22, 38–42, and 156–160), and these will be denoted as “time” in ANOVA analyses. These time points were chosen based on visual inspection of the effects of TEA and 4‐AP (see Figs. 4, 5) and because they correspond to the time points when distal axons showed maximum hyperexcitability (responses 18–22), maximum depression (responses 156–160), and a transition from hyperexcitability to depression (responses 38–42) in previous studies (Kim et al. 2012; Owen and Grover 2015). Together, these time points served as the “time” variable in ANOVA analyses (below).

#### Effects of TEA on distal axons

Application of TEA (10 mmol/L, *n* = 8) appeared to accelerate the normal biphasic changes during 100 Hz stimulation, with response amplitudes reaching a peak amplitude faster, then decaying more rapidly (Fig. 4A1). Comparisons of the amplitudes before and after TEA application were made with a two‐way repeated measures ANOVA with treatment (control vs. TEA) and time as the repeated measures. A significant main effect for time (*F* ratio: 41.54, *P* < 0.001) and a significant interaction between time and treatment (*F* ratio: 12.64, *P* < 0.005) were detected, but not a significant main effect for treatment (*F* ratio: 0.499, *P* > 0.5). Post hoc comparisons revealed a significant difference (*P* < 0.01) in amplitude during stimuli 38–42, with responses in TEA showing greater depression (47.6 ± 5.1%) than control (87.4 ± 9.3%).

Similar to our previous reports (Kim et al. 2012; Owen and Grover 2015), we observed biphasic changes in distal axon conduction latency (Fig. 4B1) and response half‐width (Fig. 4C1) over the course of 100 Hz HFS, with an initial small decrease followed by sustained increase, mirroring the amplitude changes. TEA application greatly enhanced the rate and magnitude of latency and half‐width increase. For latency changes, ANOVA detected a significant main effect of time (*F* ratio: 38.47, *P* < 0.001) and treatment (*F* ratio: 24.13, *P* < 0.005), as well as a significant interaction between the two (*F* ratio: 10.93, *P* < 0.001). Post hoc comparisons revealed significantly greater latency changes after TEA application at stimuli 18–22 (control vs. TEA: 0.01 ± 0.02 msec vs. 0.84 ± 0.19 msec, *P* < 0.05), 38–42 (control vs. TEA: 0.17 ± 0.04 msec vs. 1.15 ± 0.20 msec, *P* < 0.001), and 156–160 (control vs. TEA: 0.61 ± 0.11 msec vs. 1.58 ± 0.27 msec, *P* < 0.05). Half‐width changes were similarly affected by TEA application, with two‐way repeated measures ANOVA indicating significant main effects for time (*F* ratio: 10.58, *P* < 0.01) and treatment (*F* ratio: 62.99, *P* < 0.001), as well as a significant interaction between time and treatment (*F* ratio: 6.43, *P* < 0.005). Post hoc analysis detected significant enhancements of half‐width changes at stimuli 18–22 (control vs. TEA: −0.05 ± 0.04 msec vs. 1.21 ± 0.07 msec, *P* < 0.0001) and 38–42 (control vs. TEA: 0.15 ± 0.04 msec vs. 1.39 ± 0.17 msec, *P* < 0.0005). In summary, TEA enhanced the rate and magnitude of distal axon excitability depression (as indicated by amplitude and conduction latency changes) during HFS, and TEA also enhanced the normal increases in response half‐widths during HFS.

#### Effects of TEA on proximal axons

Unexpectedly, TEA application had minimal effects on proximal Schaffer collateral responses during HFS. There were no apparent effects of TEA on response amplitude (Fig. 4A2) or latency changes (Fig. 4B2). However, TEA appeared to reduce the rate of change in half‐width for proximal axon responses, opposite to its effect on distal axon half‐widths (compare Fig. 4C1 and C2). ANOVA of the proximal axon response amplitudes revealed a significant main effect for time (*F* ratio: 170.23, *P* < 0.001) and a significant interaction between time and treatment (*F* ratio: 3.686, *P* < 0.05), but no significant differences between control and TEA were detected in the post hoc analysis (*P* > 0.08 for all). Similarly, for latency and half‐width changes, there were significant main effects for time (latency: *F* ratio: 84.62, *P* < 0.001; half‐width: *F* ratio: 19.36, *P* < 0.001), and significant interactions between time and treatment (latency: *F* ratio: 7.53, *P* = 0.001; half‐width: *F* ratio: 19.36, *P* < 0.005). Post hoc comparisons of the proximal responses revealed no significant effects of TEA on latency changes at any time point, however, TEA did significantly reduce the change in half‐widths during stimuli 3–7 (control vs. TEA: 0.30 ± 0.05 msec vs. −0.10 ± 0.05 msec, *P* < 0.005). In summary, TEA had at best minimal effects on proximal axon responses during 100 Hz HFS.

#### Effects of 4‐AP on distal axons

Compared with TEA, 100 *μ*mol/L 4‐AP (*n* = 6) had less dramatic effects on distal Schaffer collateral excitability (compare Figs. 4, 5). Depression of response amplitudes during HFS was greater after application of 4‐AP (Fig. 5A1), with ANOVA showing significant main effects for time (*F* ratio: 55.096, *P* < 0.001) and treatment (*F* ratio: 7.23, *P* < 0.05). However, post hoc analysis revealed a significant effect of 4‐AP on distal response amplitudes only during stimuli 156–160 (control vs. 4‐AP: 45.22 ± 4.98% vs. 28.05 ± 4.55%, *P* < 0.05). Like TEA, 4‐AP also enhanced the increase in response latency (Fig. 5B12). Similar to the amplitude data, ANOVA revealed significant main effects for time (*F* ratio: 73.02, *P* < 0.001) and treatment (*F* ratio: 19.58, *P* < 0.01). Post hoc analysis indicated that 4‐AP caused a significant increase in latency during stimuli 18–22 (control vs. 4‐AP: ‐0.05 ± 0.03 msec vs. 0.40 ± 0.11 msec, *P* < 0.05), and stimuli 156–160 (control vs. 4–AP: 0.98 ± 0.05 msec vs. 1.2 ± 0.05 msec, *P* < 0.05). Likewise, 4‐AP application caused greater increases in half‐width during HFS (Fig. 5C1), with ANOVA showing significant main effects for time (*F* ratio: 7.67, *P* < 0.05) and treatment (*F* ratio: 29.07, *P* < 0.005). Post hoc analysis indicated a significant effect of 4‐AP on half‐widths only during stimuli 3–7 (control vs. 4‐AP: −0.110 ± 0.03 msec vs. 0.79 ± 0.18 msec, *P* < 0.05). In summary, 4‐AP enhanced the rate and magnitude of distal axon excitability depression (as indicated by amplitude and conduction latency changes) during HFS; 4‐AP also enhanced the normal increases in response half‐widths during HFS.

#### Effects of 4‐AP on proximal axons

As with TEA, 4‐AP did not appear to alter proximal axon response amplitudes (Fig. 5B1), latency changes (Fig. 5B2), or half‐widths (Fig. 5B3). Repeated measures ANOVA confirmed this impression. There were significant main effects for time on response amplitudes (*F* ratio: 49.56, *P* < 0.001) and changes in half‐width (*F* ratio: 8.55, *P* < 0.005), but not for changes in latency; there were no other significant main effects of time, nor any significant interaction for amplitude, latency or half‐width changes. Post hoc analysis did not reveal any significant differences between control and 4‐AP at any of the four time points tested.

In summary, blocking K_V_ channels with TEA or 4‐AP promoted the depression of excitability (reduced response amplitude accompanied by increased response latency) during HFS in distal portions of Schaffer collaterals. In contrast, the excitability of proximal Schaffer collateral axons during HFS was not affected by either channel blocker. Similarly, both channel blockers enhanced the half‐width increases during HFS in distal, but not proximal axons. The lack of effect of TEA and 4‐AP on proximal axon excitability changes during HFS does not reflect a lack of sensitivity to these blockers, since both blockers had significant effects of proximal axon responses evoked at low frequency.

### Nonspecific block of K_V_ channels enhanced distal and proximal Schaffer collateral excitability depression during burst stimulation

Because CA3 neurons typically fire in short bursts rather than long high‐frequency trains (Kandel and Spencer 1961; Ranck 1973), we examined the role of K_V_ channels blockers during burst stimulation at a 200‐msec interval (frequency of 5 Hz, or theta). For statistical analysis, we compared response amplitudes, latency changes, and half‐width changes at three time points: the first burst (stimuli 1–4), the 11th burst (stimuli 41–44), and the final burst (stimuli 157–160). These time points were chosen to assess effects of channel blockers at early, intermediate, and late points during burst stimulation. Because burst stimulation has local, within‐burst effects, as well as cumulative, across‐burst effects (Owen and Grover 2015), we conducted separate repeated measures ANOVAs at each of the three time points, comparing effects of blocker (treatment) and position (1–4) within the burst.

#### Effects of TEA on distal axons

**Figure 6:**
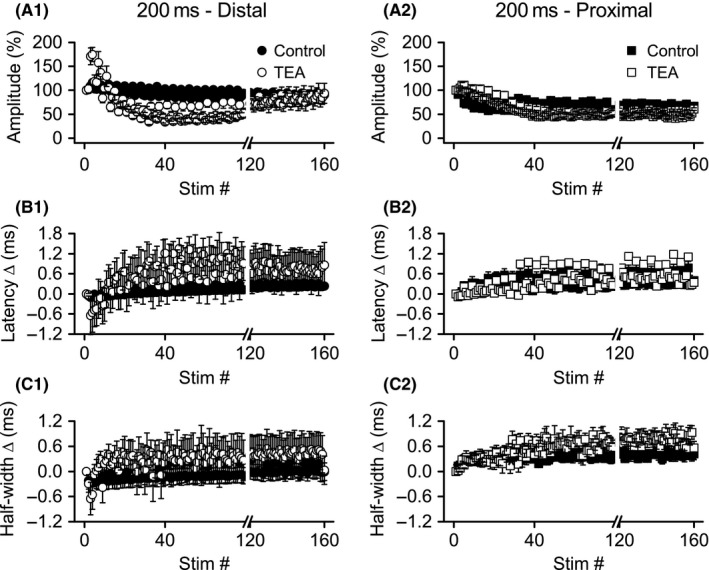
Application of TEA caused more rapid depression of distal, but not proximal, Schaffer collateral responses during theta frequency burst stimulation. Simultaneous recordings of distal (left) and proximal (right) Schaffer collateral responses during HFS were made in DNQX (30 *μ*mol/L) + CGP‐37849 (5 *μ*mol/L) before (Control) and after addition of TEA (10 mmol/L). Mean response amplitudes (A), latency changes (B), and half‐widths (C) were measured during burst stimulation (*n* = 8): distal responses showed more rapid depression, but recovered later during stimulation; proximal responses were minimally affected. Data from bursts 1 (stimuli 1–4), 11 (stimuli 41–44), and 40 (stimuli 157–160) are shown in greater detail in Figure 7. Data are not shown for stimuli 80–119 (break in *x*‐axis). HFS, high‐frequency stimulation

**Figure 7:**
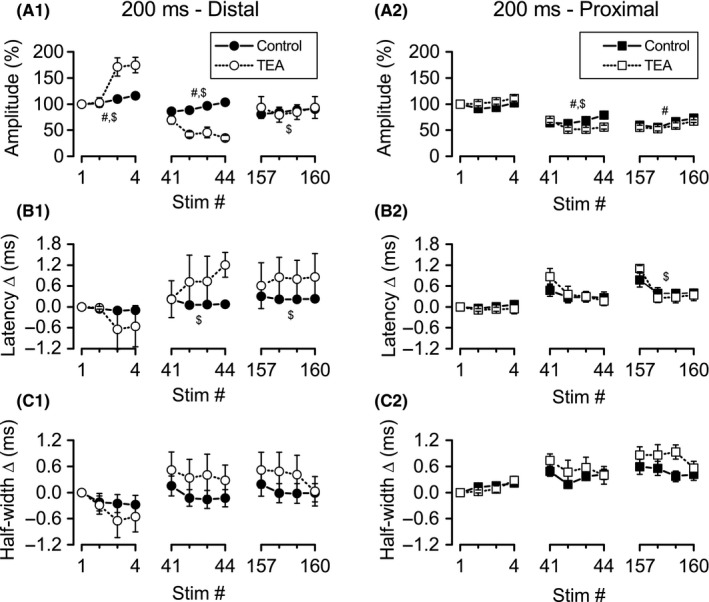
Greater effect of TEA on distal versus proximal responses during bursts 1, 11, and 40 of theta frequency burst stimulation. Simultaneous recordings of distal (left) and proximal (right) Schaffer collateral responses during high‐frequency stimulation were made in DNQX (30 *μ*mol/L) + CGP‐37849 (5 *μ*mol/L) before (Control) and after addition of TEA (10 mmol/L). Mean response amplitudes (A), latency changes (B), and half‐widths (C) were measured during bursts 1 (stimuli 1–4), 11 (stimuli 41–44), and 40 (stimuli 157–160). ^#^significant main effect of TEA (*P* < 0.05); ^$^significant interaction of TEA with stimulus position within burst (*P* < 0.05).

Distal axon response amplitudes (*n* = 8) during the first burst (stimuli 1–4) were significantly increased after TEA application (Figs. 6A1, 7A1), with ANOVA indicating a significant main effect for treatment (*F* ratio: 6.10, *P* < 0.05). ANOVA also revealed a significant main effect for stimulus position (1–4) within the burst (*F* ratio: 13.28, *P* < 0.005) as we reported previously (Owen and Grover 2015). In addition, there was a significant interaction between treatment and stimulus position (*F* ratio: 4.95, *P* < 0.05), reflecting an increase in amplitude in TEA compared to control (Figs. 6A1, 7A1). Distal axon response amplitudes were also significantly altered by TEA at burst 11 (stimuli 41–44); however, at this time point, amplitudes were reduced, rather than increased, by TEA (significant main effect for treatment, *F* ratio: 106.97, *P* < 0.001). There was not a significant main effect for stimulus position with the burst at this time point. However, there was again a significant interaction between treatment and stimulus position (*F* ratio: 9.44, *P* < 0.001), with response amplitudes progressively increasing in the control condition but decreasing in the presence of TEA (Fig. 7A1). As stimulation was continued from burst 11–40, there was a slow and progressive recovery in response amplitudes in the presence of TEA, whereas control amplitudes remained constant over the same time (Fig. 6A1). During burst 40 (stimuli 157–160), ANOVA failed to find any significant main effects, but a significant interaction between treatment and stimulus position (*F* ratio: 10.30, *P* < 0.001); as during burst 11, the significant interaction reflects an increase in response amplitudes in the control condition, compared to a decrease after TEA treatment (Fig. 7A1).

Effects of TEA on distal axon latency changes (Figs. 6B1, 7B1) appeared to mirror the effects on amplitudes, although ANOVA revealed significant effects only for burst 11 and 40. For burst 11 (stimuli 41–44), there was a significant main effect for stimulus position (*F* ratio: 3.37, *P* < 0.05) and a significant interaction between stimulus position and treatment (*F* ratio: 5.04, *P* < 0.01), reflecting decreased latencies in control conditions, but increased latencies in TEA (Fig. 7B1). The same results were obtained for burst 40 (stimuli 157–160): there was a significant main effect for stimulus position (*F* ratio: 4.44, *P* < 0.05), and a significant interaction between stimulus position and treatment (*F* ratio: 7.47, *P* < 0.005). For burst 40, as for burst 11, the significant interaction reflected a decrease in latency during the burst in the absence of TEA, compared to an increase in latency when TEA was present (Fig. 7B1). Although distal axon half‐widths appeared to be affected similar to latencies by TEA treatment (Fig. 5C1), ANOVA revealed a significant main effect for stimulus position only within burst 40 (stimuli 157–160; *F* ratio: 3.44, *P* < 0.05), with half‐widths decreasing across the burst for both control and TEA conditions (Fig. 5C1).

In summary, block of K_V_ channels by TEA led to a more rapid depression of distal axon excitability during burst stimulation, with response amplitudes significantly depressed at burst 11. Curiously, this effect of TEA dissipated with repeated stimulation, with little difference between TEA and control at burst 40. In addition, TEA altered the normal pattern of excitability change within individual bursts (an increase in excitability, as indicated by increased response amplitudes along with decreased response latencies), so that by burst 11, distal axon excitability decreased within individual bursts (decreased amplitudes along with increased latencies).

#### Effects of TEA on proximal axons

Proximal axon responses (*n* = 8) during burst stimulation, like those recorded during HFS, were less affected by TEA than distal axon responses (compare distal and proximal in Figs. 6, 7). However, ANOVA revealed a significant main effect of TEA on response amplitudes during bursts 11 and 40. For burst 11 (stimuli 41–44), there were significant main effects for stimulus position (*F* ratio: 5.07, *P* < 0.05) and TEA treatment (*F* ratio: 32.07, *P* = 0.001), as well as a significant interaction between these two variables (*F* ratio: 7.80, *P* < 0.01). The main effect for TEA treatment indicates smaller response amplitudes throughout the burst, and the interaction reflects opposite patterns of change within the burst, with control responses increasing in amplitude but TEA responses decreasing in amplitude (Fig. 7A2). For burst 40 (stimuli 157–160), ANOVA revealed significant main effects for stimulus position (*F* ratio: 9.68, *P* < 0.01), with responses increasing within the burst. ANOVA also revealed a significant main effect for TEA treatment (*F* ratio: 7.25, *P* < 0.05), with smaller response amplitudes after TEA application. The interaction between stimulus position and treatment was not significant for burst 40.

Analysis of latency change data revealed a significant main effect for stimulus position within bursts 11 (*F* ratio: 10.29, *P* < 0.001) and 40 (*F* ratio: 19.38, *P* < 0.001), with response latencies decreasing across stimuli within both bursts. For burst 40, there was also a significant interaction between stimulus position and TEA treatment (*F* ratio: 4.79, *P* < 0.05), reflecting a smaller decrease in latency in the control condition compared to TEA (Fig. 7B2). Analysis of half‐width changes revealed only a significant main effect for stimulus position during burst 1 (*F* ratio: 11.98, *P* < 0.001), with equal increases in half‐widths across the burst for both control and TEA.

To summarize, TEA had less dramatic effects on proximal axons during burst stimulation compared to distal axons. In addition, whereas TEA application decreased distal axon excitability, both across bursts and within bursts, TEA had no consistent effect on proximal axon excitability within bursts. For example, TEA‐treated proximal axons showed decreased response amplitudes within burst 11 (suggesting decreased excitability), but also showed decreased response latencies (suggesting increased excitability).

#### Effects of 4‐AP on distal axons

**Figure 8:**
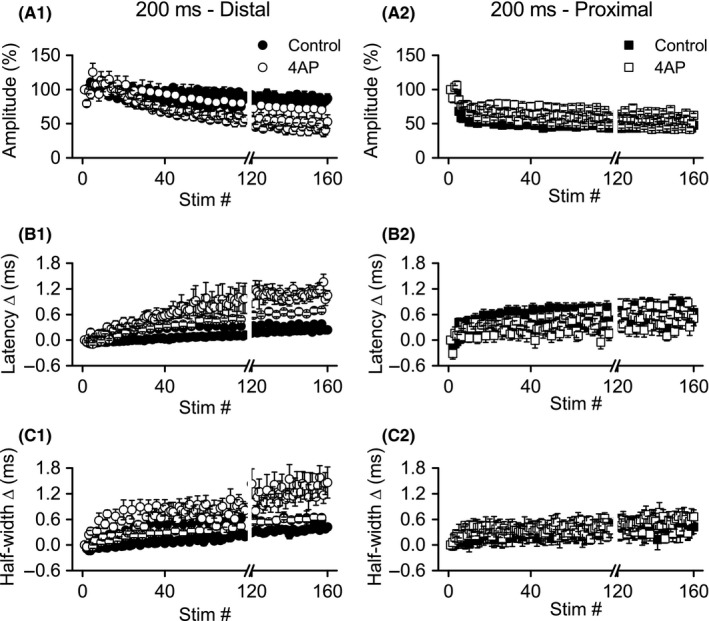
Application of 4‐AP caused more rapid depression of distal, but not proximal, Schaffer collateral responses during theta frequency burst stimulation. Simultaneous recordings of distal (left) and proximal (right) Schaffer collateral responses during high‐frequency stimulation were made in DNQX (30 *μ*mol/L) + CGP‐37849 (5 *μ*mol/L) before (Control) and after addition of 4‐AP (100 *μ*mol/L). Mean response amplitudes (A), latency changes (B), and half‐widths (C) were measured during burst stimulation (*n* = 6): distal responses showed more rapid depression; proximal responses were minimally affected. Data from bursts 1 (stimuli 1–4), 11 (stimuli 41–44), and 40 (stimuli 157–160) are shown in greater detail in Figure 9. Data are not shown for stimuli 80–119 (break in *x*‐axis).

**Figure 9:**
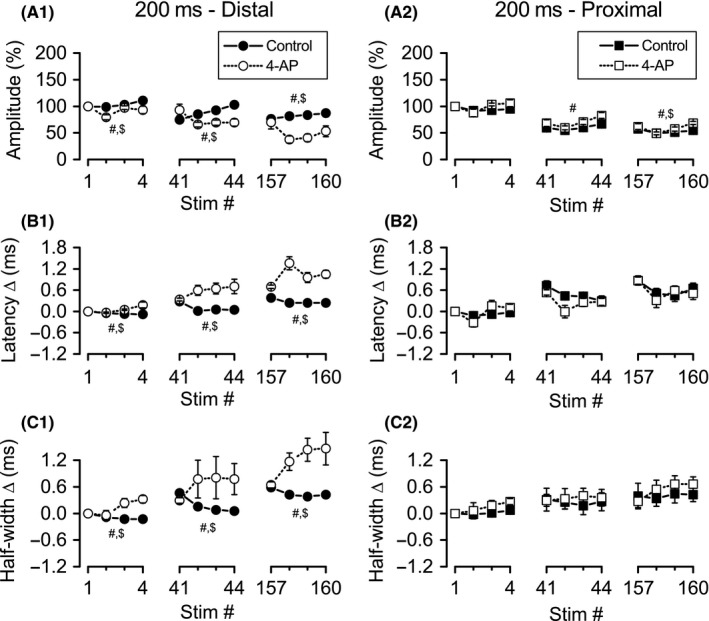
Greater effect of 4‐AP on distal versus proximal responses during bursts 1, 11, and 40 of theta frequency burst stimulation. Simultaneous recordings of distal (left) and proximal (right) Schaffer collateral responses during high‐frequency stimulation were made in DNQX (30 *μ*mol/L) + CGP‐37849 (5 *μ*mol/L) before (Control) and after addition of 4‐AP (100 *μ*mol/L). Mean response amplitudes (A), latency changes (B), and half‐widths (C) were measured during bursts 1 (stimuli 1–4), 11 (stimuli 41–44), and 40 (stimuli 157–160). ^#^Significant main effect of 4‐AP (*P* < 0.05); ^$^significant interaction of 4‐AP with stimulus position within burst (*P* < 0.05).

Unlike TEA, which increased response amplitudes during the first burst (stimuli 1–4), 4‐AP significantly reduced response amplitudes (*F* ratio: 14.50, *P* < 0.05; Figs. 8A1, 9A1; *n* = 6). There was also a significant main effect for stimulus position (*F* ratio: 7.18, *P* < 0.005) and a significant interaction between stimulus position and 4‐AP treatment (*F* ratio: 8.52, *P* < 0.005), with response amplitudes increasing from position 1–4 in controls, but decreasing after 4‐AP treatment. A similar pattern was observed during burst 11 (stimuli 41–44), with a significant main effect for 4‐AP treatment (*F* ratio: 21.70, *P* < 0.01) and a significant interaction between treatment and stimulus position (*F* ratio: 12.50, *P* < 0.001); the main effect for stimulus position did not, however, reach significance. The significant interaction during burst 11 again reflected different changes within the burst under control conditions and after 4‐AP application (Fig. 9A1). For burst 40 (stimuli 157–160), as for burst 1, there were significant main effects for 4‐AP treatment (*F* ratio: 16.56, *P* = 0.01), stimulus position (*F* ratio: 13.04, *P* < 0.001), and the interaction of treatment with stimulus position (*F* ratio: 16.44, *P* < 0.001). The significant interaction once again was due to opposite changes in response amplitudes within the burst in control and 4‐AP (Fig. 9A1).

Distal axon response latency changes (Figs. 8B1, 9B1) were significantly increased after 4‐AP treatment at all three time points: burst 1, 11, and 40 (*F* ratios: 10.50, 13.06, and 50.43; *P* < 0.05, 0.05, and 0.005; respectively). While the main effect for stimulus position was only significant for burst 40 (*F* ratio: 9.08, *P* < 0.005), the interaction between treatment and stimulus position was significant for all three time points (*F* ratios: 8.13, 10.49, and 16.45; *P* < 0.05, 0.05, and 0.001). The significant interactions again denoted opposite patterns of latency changes within individual bursts, with latencies decreasing in control conditions, but increasing in the presence of 4‐AP (Fig. 9B1). Half‐widths, like latencies, were increased by 4‐AP at all time points examined (bursts 1, 11, and 40; *F* ratios: 37.28, 15.06, and 12.04; *P* < 0.005, 0.05, and 0.05, respectively; Figs 8C1, 9C1). The main effect of stimulus position was not significant at any of the three time points, but the interaction between treatment and stimulus position was significant at all three time points (bursts 1, 11, and 40; *F* ratios: 4.88, 5.49, and 8/05; *P* < 0.05, 0.05, 0.005). For half‐width changes, as for latency changes, the significant interactions again reflected opposite patterns between control and 4‐AP conditions, with half‐widths decreasing across bursts in control, but increasing across bursts in 4‐AP.

#### Effects of 4‐AP on proximal axons

Similar to TEA, 4‐AP (*n* = 6) appeared to have minimal effects on proximal Schaffer collateral responses compared to distal responses (compare distal and proximal in Figs. 8, 9). ANOVA revealed significant main effects of 4‐AP on response amplitudes during bursts 11 (*F* ratio: 6.65, *P* = 0.05) and 40 (*F* ratio: 9.61, *P* < 0.05), with proximal response amplitudes slightly increased in the presence of 4‐AP (in contrast to distal responses, where amplitudes were reduced by 4‐AP). During burst 11, there was a significant main effect for stimulus position, with response amplitudes increasing within the burst in both control and 4‐AP conditions (*F* ratio: 23.55, *P* < 0.001; Fig. 7A2). During burst 40, there was a significant main effect for stimulus position (*F* ratio: 13.05, *P* < 0.001) and a significant interaction of stimulus position with 4‐AP treatment (*F* ratio: 4.94, *P* < 0.05), reflecting a small decrease in response amplitudes under control conditions, but a slight increase in amplitudes during the burst in 4‐AP (Fig. 7A2). For distal response latency changes, there were no significant main effects for 4‐AP treatment, nor any significant interactions between treatment and stimulus position for any of the three bursts. However, there were significant main effects for stimulus position within bursts 1 and 11 (Fig. 7B2; burst 1, *F* ratio: 6.43, *P* = 0.05; burst 11, *F* ratio: 14.14, *P* < 0.001). There were no significant main effects or interactions for any of the three bursts for proximal axon half‐widths.

In summary, as with TEA, the effects of 4‐AP on proximal axons were less pronounced than on distal axons. Moreover, 4‐AP had opposite effects on response amplitudes in proximal axons (increased amplitudes) compared to distal axons (decreased amplitudes). Like TEA, 4‐AP also altered distal axon excitability, both across bursts and within bursts. Under control conditions, although distal axon excitability decreased across bursts (decreased amplitudes and increased latencies), excitability increased within individual bursts (increased amplitudes and decreased latencies). In comparison, when distal axons were treated with 4‐AP, excitability decreased cumulatively across bursts as well as within individual bursts.

## Discussion

We previously reported that distal portions of the Schaffer collaterals undergo a period of hyperexcitability early during high‐frequency and burst stimulation, which is followed later by depression of excitability (Kim et al. 2012; Owen and Grover 2015). Proximal Schaffer collaterals, in contrast, show only depression. We hypothesized that K_V_ channel blockade would promote proximal axon hyperexcitability and abolish these differences between distal and proximal axons. Contrary to our expectation, block of K_V_ channels by either TEA or 4‐AP had virtually no effect on proximal axons, but reduced hyperexcitability and promoted depression of distal axons during high‐frequency or burst stimulation.

### Comparison to previous findings

Palani et al. (2010) demonstrated a hyperexcitable period in Schaffer collaterals that was enhanced by block of K_V_ channels. In the Palani et al. (2010) study, a low concentration (20 *μ*mol/L) of 4‐AP, and the K_V_1 blockers dendrotoxin and margatoxin, all enhanced hyperexcitability, whereas a higher concentration of 4‐AP (1 mmol/L) reduced hyperexcitability. Similarly, Lopantsev et al. (2003) reported that targeted deletion of the *Kcna1* gene that codes for the K_V_1.1 subunit resulted in enhanced excitability (burst firing) in CA3 pyramidal neurons in response to antidromic stimulation of Schaffer collaterals when extracellular K^+^ was increased to 6 mmol/L. Our finding that block of K_V_ channels reduced hyperexcitability in Schaffer collaterals might appear to contradict these two studies which both reported enhanced Schaffer collateral excitability with loss of K_V_ channel function. However, there are important differences between our study and those of Palani et al. and Lopantsev et al. which may account for the seemingly discrepant results. First, the loss of excitability that we observed was during periods of repetitive stimulations of Schaffer collaterals, but Palani et al. and Lopantsev et al. used single and paired‐pulse stimulation of the axons. These different types of stimulation would be expected to have different consequences for activation and inactivation of the various voltage‐gated ion channels present in Schaffer collaterals, with potentially different effects on excitability. Second, although the concentration of 4‐AP that we used (100 *μ*mol/L) was close to the concentration Palani et al. reported as effective for enhancing excitability, the higher concentration we used should have resulted in greater block of K_V_ channels (block of a greater fraction of the same K_V_ channel types and possible block of additional types of K_V_ channels). In other previous studies (Meeks and Mennerick 2004; Meeks et al. 2005) the effects of increasing extracellular K^+^ on Schaffer collateral function have been investigated. In these studies, elevated extracellular K^+^ caused a more rapid decrease in excitability during period of high‐frequency firing, similar to our results when K_V_ channel blockers were present. While raising extracellular K^+^ will have additional effects, one effect would be to reduce the effectiveness of K_V_ channels in causing hyperpolarization of membrane potential.

### Possible contributions of specific potassium channels

As nonselective blockers, TEA and 4‐AP have broad ranges of activity against K_V_ channels (Gutman et al. 2005). In considering which K_V_ channels might have been affected by the blockers we used, it is helpful to first review the K_V_ channels known to be localized to Schaffer collaterals. The following K^+^ channel subunits have been localized to Schaffer collateral axon initial segments, axons, or axon terminals by imaging or functional studies: K_V_1.1, 1.2, 1.4 (Monaghan et al. 2001; Palani et al. 2010; Kirizs et al. 2014), K_V_2.1 (Sarmiere et al. 2008), K_V_7.2, 7.3 (Vervaeke et al. 2006; Klinger et al. 2011), K_Ca_1.1 (Hu et al. 2001; Misonou et al. 2006; Sailer et al. 2006). While K_V_3 subunits are found in some axons and terminal (Trimmer 2015), within the hippocampus, K_V_3 subunits are predominately associated with the mossy fiber axons of dentate gyrus granule cells (Veh et al. 1995; Chang et al. 2007; Alle et al. 2011) and fast‐spiking GABAergic interneurons (Du et al. 1996; Sekirnjak et al. 1997; Martina et al. 1998; Tansey et al. 2002).

Although TEA and 4‐AP had broadly similar effects in our experiments, there were several notable differences between the two blockers. First, during low‐frequency stimulation, while both blockers caused a doubling of half‐widths in distal axons, TEA, but not 4‐AP, increased proximal axon half‐width. Second, during HFS, TEA, but not 4‐AP, strongly enhanced distal axon response depression at early time points (at stimuli 18–22 and 38–42). Third, during burst stimulation, effects of TEA on distal axon responses appeared to peak around burst 11 (stimuli 41–44), with some recovery (at least of response amplitudes) up to around burst 35–40 (stimuli 130–160). In contrast, the effects of 4‐AP on distal axon responses continued to increase up through the end of burst stimulation (burst 40, stimuli 156–160). These differences suggest that the two blockers acted on at least partially distinct sets of K^+^ channels. Considering the K^+^ channels known to be present within Schaffer collaterals, K_V_1.2 is the most likely candidate for unique effects of 4‐AP; K_V_1.2 should have been minimally affected by the TEA concentration we used (Grissmer et al. 1994; Al‐Sabi et al. 2013), but should have been partially blocked by the 4‐AP concentration we used (Po et al. 1993; Grissmer et al. 1994). Unique effects of TEA could have been mediated by K_V_1.1, K_V_2, K_V_7, or K_Ca_1.1, all of which may be at least partially blocked by the TEA concentration we used (Frech et al. 1989; Stühmer et al. 1989; Lang and Ritchie 1990, Taglialatela et al. 1991; Po et al. 1993; Grissmer et al. 1994; Shieh and Kirsch 1994; Shen et al. 1994; Gòmez‐Hernandez et al. 1997; Gutman et al. 2005; Quinn and Begenisich 2006; Tang et al. 2010; Al‐Sabi et al. 2013), but should be insensitive to the 4‐AP concentration we used (Frech et al. 1989; Stühmer et al. 1989; Kirsch et al. 1993; Grissmer et al. 1994; Gutman et al. 2005; Tang et al. 2010). Our suggestion that block of K_V_1.2 by 4‐AP is responsible for enhanced depression of excitability may seem at odds with the conclusions of Palani et al. (2010) that block of K_V_1.2 causes hyperexcitability, but as discussed above, there are substantial differences in the stimulation pattern (high‐frequency and burst stimulation vs. single and paired stimulation) and quantity (160 stimuli vs. 1 or 2 stimuli) between this study and Palani et al. (2010). These differences in stimulation could allow the same K_V_ channel type to maintain excitability under some conditions (e.g. by repolarizing the axon membrane potential during high‐frequency and burst stimulation), but limit excitability under others (e.g. by generating an afterhyperpolarization following a single action potential).

### Physiological relevance and implications

Our present and past (Kim et al. 2012, Owen and Grover 2015) findings and the findings of others (reviewed above) indicate that Schaffer collaterals may become unreliable conductors of action potentials during periods of high activity, but changes in reliability are dependent on the nature (pattern, frequency and duration) of activity. Moreover, while K_V_ channels help maintain reliability during long periods of sustained high frequency or burst stimulation (this paper), K_V_ channels limit hyperexcitability during single or paired stimulation (Lopantsev et al. 2003 and Palani et al. 2010). Decreased reliability of conduction during sustained high‐frequency stimulation affects information transfer as well as physiological plasticity (LTP) at Schaffer collateral‐CA1 synapses (Grover et al. 2009, Kim et al. 2010).

In addition to “global” changes in excitability across repeated high‐frequency and burst stimulation, there are more “local” changes in excitability that occur within single stimulus bursts (as shown here and also in Owen and Grover 2015). Curiously, this effect of stimulus position within a burst is not stationary, but varies with the number of bursts previously given. These complex patterns of excitability change most likely reflect altered availability of specific ion channel types at different times during burst stimulation. For example, within any single burst, K_V_ channel inactivation is likely to accumulate with repeated stimulation, but some degree of recovery from inactivation will occur between bursts. If the interval between bursts is insufficient for complete recovery, then inactivation will accumulate across bursts as well as within bursts. Inactivation will vary among Na_V_ and the multiple K_V_ channel types that contribute to Schaffer collateral excitation. Like “global” changes in excitability, these complex “local” changes in excitability may also affect information transfer and plasticity.

### Possible factors contributing to different sensitivity to potassium channel blockers in proximal and distal Schaffer collaterals

In this study, we observed striking differences in the effects of TEA and 4‐AP on distal compared to proximal Schaffer collaterals. Surprisingly, during periods of intense activity (high‐frequency and burst stimulation) proximal axons were largely resistant to the blockers, whereas distal axon function was profoundly altered. Two factors are likely to contribute to this differing sensitivity. First, differences in localization and density of voltage‐dependent ion channels. These differences have been studied primarily at the axon initial segment, the most proximal portion of the axon. The axon initial segments of CA3 pyramidal neurons are known to be enriched in Na_V_1.6 (Lorincz and Nusser 2008), K_V_2.1 (Sarmiere et al. 2008), and K_V_7.2/7.3 (Klinger et al. 2011). A greater diversity or density of ion channel types might make proximal axons more resistant to blockers. Second, Schaffer collaterals vary with distance from the cell body in density of axon branch points and axon diameters (Ishizuka et al. 1990; Li et al. 1994; Wittner et al. 2007). These morphological differences might make distal axons more sensitive to block of K_*V*_ channels. Of course, both of these factors could interact to increase the sensitivity of distal Schaffer collaterals to K_V_ channel blockade.

## Conflict of Interest

None of the authors declare a conflict of interest.
